# Neuro–immune interactions in colorectal cancer: mechanistic insights and therapeutic implications within the tumor immune microenvironment

**DOI:** 10.3389/fimmu.2026.1852495

**Published:** 2026-08-21

**Authors:** Hongbin Zhen, Chunxia Wang, Xia Wang, Huanyuan Huang, Jun Li, Meie Zhao

**Affiliations:** 1 Department of Clinical Laboratory, The First People Hospital of Lanzhou, Lanzhou, Gansu, China; 2 Department of Examination Management, Chongqing Technical Center for Drug Evaluation & Inspection, Chongqing, China

**Keywords:** brain–gut axis, cancer neuroscience, colorectal cancer, enteric nervous system, immunotherapy, neuro–immune crosstalk, neuromodulation, tumor immune microenvironment

## Abstract

Within the CRC microenvironment, neural and immunological systems constitute a regulatory axis with strong dynamism and bidirectionality, thereby profoundly influencing tumor initiation, immune evasion, and therapeutic resistance. Here, we systematically outline the anatomical as well as functional underpinnings, the molecular mechanisms, and the translational potential of neuro–immune crosstalk within CRC. A unique biological context is offered by the gut, which houses two key components: an autonomous “second brain”, namely the enteric nervous system (ENS), and the human body’s largest immune organ. Consequently, this arrangement provides a natural platform supporting intensive neuro–immune crosstalk. At the central level, the classical brain–gut axis provides the physiological framework through which neuroendocrine and autonomic signals regulate intestinal and systemic immunity. Tumor-derived signals may functionally rewire this pre-existing network, thereby establishing a tumor–brain–immune circuit characterized by reciprocal signaling and positive-feedback amplification. In the periphery, nerve fibers of sympathetic, parasympathetic, and sensory origins regulate immune cell functions via neurotransmitters and neuropeptides—including norepinephrine, acetylcholine, CGRP, and substance P—which in turn drives immune suppression or tolerance. By contrast, neuronal activity and plasticity are modulated by immune cells through the production of neurotransmitter-like molecules and cytokines including IL-17A. In this process, glial cells—Schwann cells among them—serve as critical intermediaries. Given this context, emerging therapies directed against the neuro–immune axis include repurposed β-adrenergic blockers and neuropeptide receptor antagonists, together with neuromodulatory interventions such as vagus nerve stimulation and microbiota-based approaches. These therapies are promising avenues to overcome resistance to immunotherapy and to remodel the immune microenvironment of the tumor. It will be necessary for future investigations to integrate multi-omics profiling with precise neuromodulation technologies, so as to construct high-resolution maps of neuro–immune interactions and to advance interventions towards nerve subtype–specific precision. Ultimately, this effort will shift CRC treatment paradigms from immune precision toward neuro–immune precision.

## 1 Introduction

Over the course of evolution, the nervous and immune systems have co-evolved cooperative defense strategies, sharing sensory capabilities and signaling molecules to counter environmental threats (1, 2). Although traditionally viewed as largely separate systems, a growing body of evidence now indicates that extensive bidirectional crosstalk between neural and immune pathways plays a decisive role in chronic inflammation, autoimmune diseases, and cancer progression (3, 4). As a result, neuroimmunology has become a key interdisciplinary field at the forefront of modern tumor biology and immunology.

Within this conceptual framework, the rapidly advancing discipline of cancer neuroscience has firmly established that the nervous system does not simply serve as a passive element within the tumor microenvironment (TME) (5, 6). Rather, tumor-associated nerves actively drive cancer progression and immune evasion via neural invasion, neurotransmitter release, and neuropeptide-mediated signaling (7). Conversely, immune cells reciprocally influence neural activity, creating integrated neuro–immune–tumor regulatory circuits that fundamentally reshape antitumor immunity (8, 9).

Colorectal cancer (CRC) offers an especially well-suited model for studying neuro-immune crosstalk (10, 11). The gastrointestinal tract harbors the enteric nervous system (ENS), often called the “second brain”, while simultaneously serving as the body’s largest immune organ, constantly interacting with commensal microbes and dietary antigens (12, 13). In this setting, nerve endings, immune cells, and epithelial cells reside in close physical proximity, forming a permissive environment for neuro-immune communication (14, 15). Moreover, the gut microbiota constitutes a third regulatory layer, shaping both neural signaling and immune responses through metabolites and pattern-recognition pathways. Disruption of this tripartite balance can promote tumorigenesis and facilitate immune escape.

From a clinical standpoint, the neuro-immune dimension carries substantial implications. Although immune checkpoint inhibitors (ICIs) have revolutionized the treatment of several malignancies, most patients with microsatellite-stable CRC still derive limited benefit. Beyond tumor-intrinsic and immune-intrinsic factors, neural regulation may act as an upstream driver that renders the CRC microenvironment immunologically “cold”. Increased nerve density and neural invasion correlate with poor prognosis in CRC; nevertheless, how neural signals systematically reprogram antitumor immunity, how distinct neural pathways exert divergent effects, and how these processes affect therapeutic responses remain incompletely understood (16). However, the disease specificity of the available evidence varies substantially. Throughout this Review, we distinguish direct evidence obtained from CRC tissues or experimental CRC models from indirect evidence derived from non-malignant intestinal conditions and cross-tumor evidence obtained from other cancer types. The latter categories provide biologically plausible hypotheses but should not be interpreted as established CRC-specific mechanisms without functional validation in colorectal tumor models.

Accordingly, in this Review, we comprehensively discuss neuro-immune interactions relevant to CRC, integrating their anatomical underpinnings, molecular signaling networks and regulatory logic.

## 2 Anatomical and functional foundations of the neuro–immune microenvironment in colorectal cancer

### 2.1 Central regulatory layer: the brain–gut axis and bidirectional feedback

**Figure 1:**
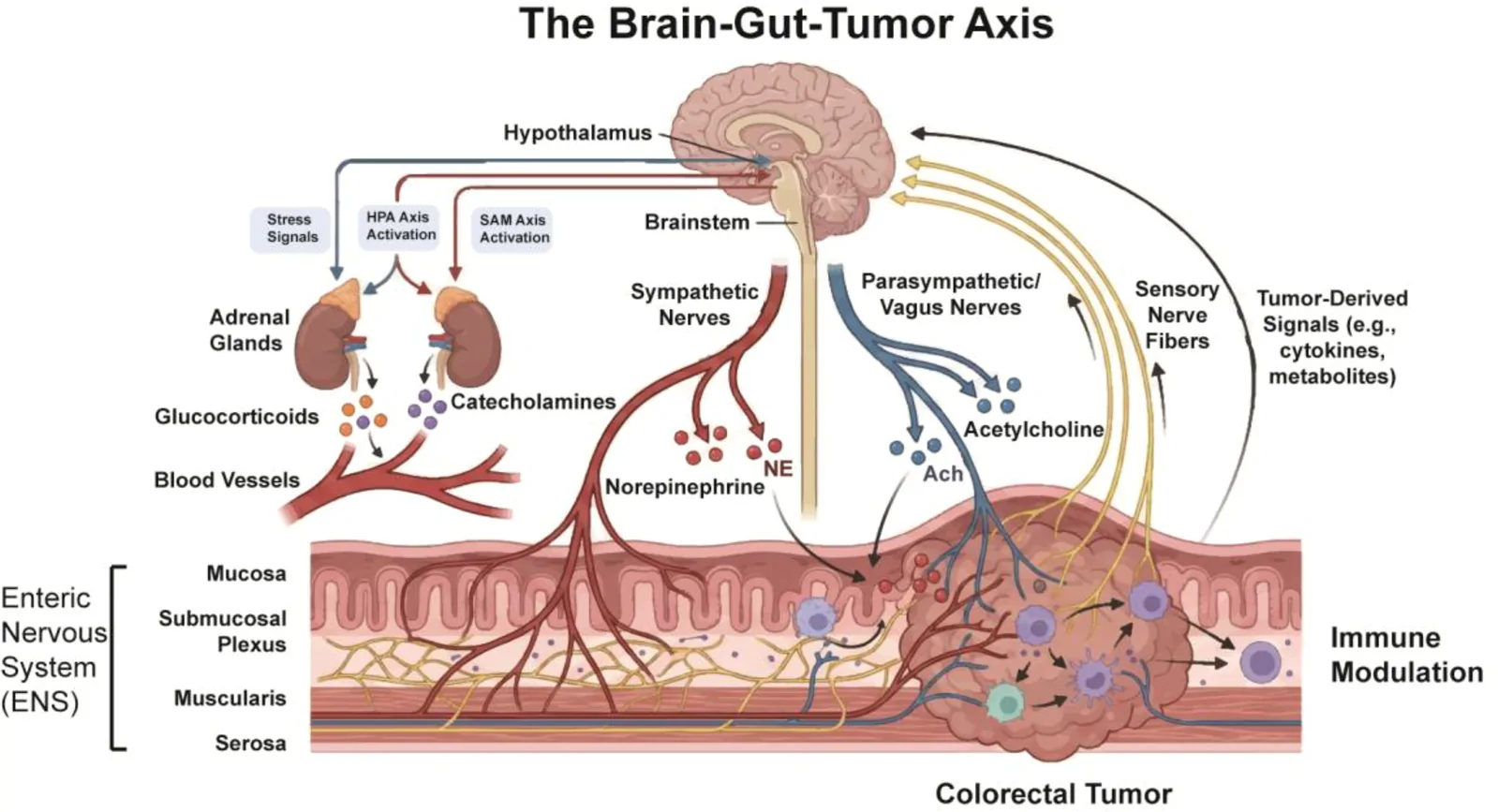
Bidirectional brain–gut–tumor neuro-immune axis in colorectal cancer. The central nervous system communicates with the gut and colorectal tumor microenvironment through the hypothalamic–pituitary–adrenal (HPA) axis, autonomic nervous system (sympathetic and parasympathetic pathways), and sensory neural circuits. Stress-associated neuroendocrine and neural signals propagate downward to modulate local immune states, whereas tumor- and gut-derived signals feed back to the brain via neural and humoral routes. The enteric nervous system (ENS) functions as a local integrative hub, coordinating central neural inputs with tumor-associated immune regulation. Created in BioRender. Zhao, M. (2026) https://BioRender.com/l9tuygw.

A core communication pathway linking the CNS to intestinal functions is represented by the brain–gut axis (17, 18). Depicted in **Figure 1** is the macroscopic organization of this bidirectional brain–gut–tumor neuro-immune axis within colorectal cancer. Psychological stress, systemic inflammatory signals, and tumor burden become translated into systemic outputs of glucocorticoids and catecholamines via activation of both the hypothalamic–pituitary–adrenal (HPA) axis and the sympathetic–adrenal–medullary axis (19, 20). In the setting of CRC, sustained elevations of these neuroendocrine responses broadly suppress the antigen-presenting capacity of dendritic cells and reduce the cytotoxic activity of CD8^+^ T cells together with natural killer (NK) cells, thereby systemically fostering immune suppression and tolerance (21, 22).

This regulatory axis is not unidirectional (23, 24). The classical brain–gut axis provides the broader physiological framework through which central neural, neuroendocrine, autonomic and intestinal signals are exchanged (17, 18). Tumor-derived and tumor-associated signals may recruit and functionally rewire this pre-existing network, thereby generating a tumor–brain–immune regulatory circuit (5, 6, 11, 25, 26). Conceptually, this circuit should not be regarded as an anatomical axis separate from or parallel to the brain–gut axis. Rather, it represents a tumor-driven subcircuit nested within the broader brain–gut architecture. Its defining features include tumor-initiated signaling, reciprocal communication, positive-feedback amplification and sustained remodeling of the tumor microenvironment (5, 6, 11).

### 2.2 Peripheral effector layer: remodeling of the enteric nervous system and tumor-associated innervation

CRC develops within a uniquely specialized neural anatomical context (12). Within the gastrointestinal tract resides a highly complex and relatively autonomous enteric nervous system (ENS), which consists of the myenteric and submucosal plexuses. Beyond its independent coordination of intestinal motility and secretion, the ENS stays in intimate spatial proximity to immune cells and plays a critical part in preserving immune homeostasis within the intestine (27).

Substantial remodeling of both the ENS and the intratumoral neural architecture occurs during tumor progression. Marked alterations are observed in the density and spatial arrangement of sympathetic, parasympathetic, and sensory nerve fibers, and these alterations correlate with tumor stage as well as clinical prognosis (28, 29). Sympathetic nerves primarily exert their effects by releasing norepinephrine; parasympathetic fibers signal through acetylcholine; and sensory nerves secrete neuropeptides such as calcitonin gene-related peptide (CGRP) and substance P. Each of these mediates distinct immunomodulatory functions (30, 31).

Two sources give rise to tumor-associated nerves: functional reprogramming of pre-existing neural fibers and tumor-induced axonogenesis (32). The latter process is largely driven by neurotrophic factors secreted by CRC cells together with stromal components. For instance, nerve growth factor and brain-derived neurotrophic factor activate Trk and p75^NTR^ receptor signaling pathways, actively guiding nerve fibers toward tumor regions and promoting their infiltration (33). This “neural recruitment” process establishes novel communication channels between tumor cells and immune cells within the tumor microenvironment (5).

### 2.3 Molecular communication bridges: immune regulatory networks mediated by neurotransmitters, neuropeptides, and neurotrophic factors

**Figure 2:**
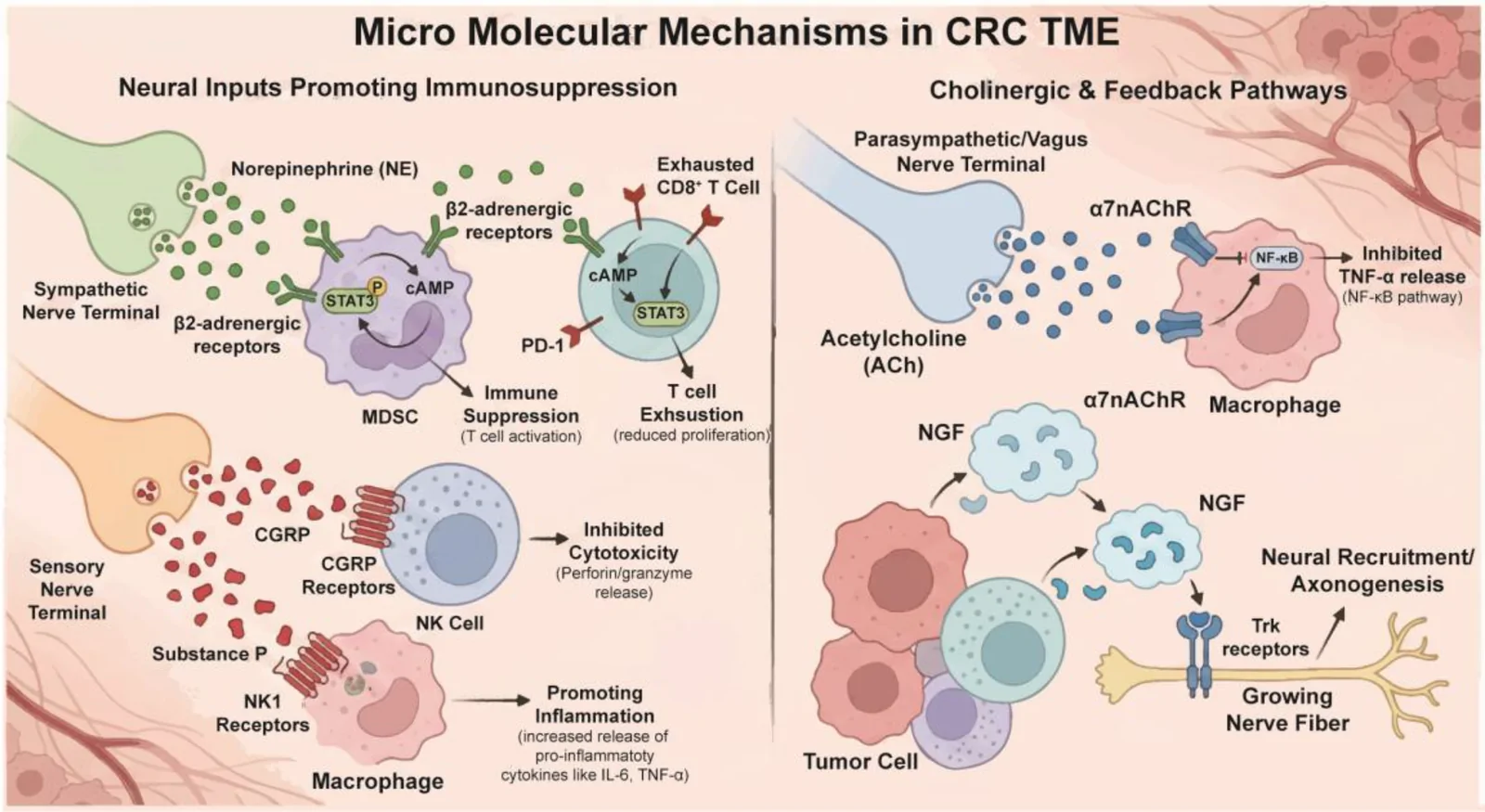
Molecular mechanisms of neuro–immune crosstalk shaping immunosuppression in the colorectal cancer tumor microenvironment. Sympathetic and sensory nerve–derived mediators, including norepinephrine, calcitonin gene–related peptide (CGRP), and substance P, engage cognate receptors on immune cells to promote myeloid-driven immunosuppression, T cell exhaustion, and impaired cytotoxic function. In contrast, parasympathetic cholinergic signaling via acetylcholine and α7 nicotinic acetylcholine receptors dampens excessive inflammation. Tumor- and immune-derived neurotrophic factors, such as nerve growth factor (NGF), further reinforce neural recruitment and axonogenesis, establishing a feedforward neuro–immune regulatory loop within the tumor microenvironment. Created in BioRender. Zhao, M. (2026) https://BioRender.com/pfur5pm.

Neural regulation of the CRC immune microenvironment is ultimately executed through the release and recognition of a range of specific molecular mediators. **Figure 2** provides a schematic summary of the major molecular mediators and signaling pathways that underlie neuro–immune crosstalk within the colorectal cancer tumor microenvironment. Classical neurotransmitters—including norepinephrine and epinephrine—act on T cells, dendritic cells, macrophages, and other immune cell populations via α- and β-adrenergic receptors, generally driving immunosuppressive and exhaustion-associated programs (21). Through cholinergic receptors, acetylcholine participates in the so-called “cholinergic anti-inflammatory pathway”, with its immunoregulatory effects being highly dependent on context (34).

Neuropeptides released from sensory nerves, such as CGRP, substance P, and vasoactive intestinal peptide, bind to their respective receptors and either suppress effector immune cell functions or promote regulatory phenotypes, thereby shaping antitumor immune responses (35). Furthermore, classical neurotransmitters with immunomodulatory functions—including γ-aminobutyric acid (GABA), serotonin and dopamine—can influence immune-cell activation, differentiation and functional polarization (36–40). GABA is a classical inhibitory neurotransmitter rather than a non-classical neural mediator. Nevertheless, the cellular sources and functional importance of these neurotransmitters in the CRC microenvironment remain incompletely defined.

Within this regulatory network, neurotrophic factors play a dual role. Not only are they key drivers of neural remodeling, but they also directly modulate immune cell function in a receptor-dependent manner, further blurring the functional boundaries between the nervous and immune systems (41). Taken together, these mediators form a dense and interconnected signaling network that constitutes the molecular foundation for neuro–immune crosstalk in the colorectal cancer microenvironment.

## 3 Neural regulation of antitumor immunity in colorectal cancer

Within the CRC microenvironment, distinct nerve fiber types continuously reshape both the functional status and spatial organization of immune cells via released neurotransmitters and neuropeptides (42, 43). Depending on the neural subtype involved, the targeted immune cell population, and the specific tumor progression stage, the immunological outcomes of sympathetic, parasympathetic, and sensory neural signaling vary considerably. **Table 1** summarizes the principal neural pathways, their key mediators, immune targets, and functional consequences in colorectal cancer. A systematic overview of the immunoregulatory mechanisms driven by these major neural pathways is provided below.

**Table 1:** Neural pathways potentially involved in immune regulation in colorectal cancer.

Neural subtype	Key mediators	Immune cell targets	Dominant signaling	Immunological outcome	CRC-specific notes	Evidence context
Sympathetic nerves	NE, Epi	CD8^+^ T, MDSCs, Tregs	β2-AR–cAMP–STAT3	T cell exhaustion, MDSC expansion	Correlates with nerve density & poor prognosis	CRC and pan-cancer evidence (45–47, 64)
Parasympathetic (vagal)	ACh	Macrophages, T cells	α7nAChR–NF-κB	Context-dependent immune suppression	Stage-specific effects in CRC	Intestinal non-cancer evidence (49)
Sensory nerves	CGRP	CD8^+^ T, NK cells	cAMP–PKA	Reduced cytotoxicity	Enriched in tumor margin	Non-CRC tumor evidence (52)
Sensory nerves	Substance P	DCs, macrophages	NK1R–NF-κB	PD-L1 upregulation	Links inflammation & tolerance	Non-CRC/inflammatory evidence (53)
ENS-derived neurons	5-HT, GABA	Myeloid cells	GPCR signaling	Immune modulation	Gut-specific neural layer	Pan-cancer evidence; CRC limited (37–40)
Tumor-induced axons	NGF, BDNF	Multiple	Trk–MAPK	Neural remodeling	Drives neuro–immune coupling	Mainly non-CRC evidence (33, 41, 55)

### 3.1 Sympathetic signaling: a central driver of immunosuppression

Among neural pathways implicated in tumor regulation, the sympathetic nervous system has received the most extensive investigation. In malignancy, increased sympathetic nerve fiber density together with enhanced norepinephrine signaling correlates closely with the establishment of an immunosuppressive tumor microenvironment (44). Through binding predominantly to β-adrenergic receptors expressed on immune cells, norepinephrine and epinephrine exert their effects. Among these receptors, β2-adrenergic receptors (β2-AR) are abundantly present across many immune cell types, and their activation markedly improves both the survival and the suppressive capacity of myeloid-derived suppressor cells (MDSCs) (45).

Metabolic reprogramming is induced in MDSCs by β-AR signaling, promoting fatty acid oxidation to meet the energy demands required for their immunosuppressive activity (46). At the same time, STAT3 pathway activation upregulates the expression of suppressive molecules such as ARG1 and PD-L1 (45). At the T cell level, sustained β-AR signaling directly drives exhaustion of CD8^+^ T cells, which is characterized by increased expression of inhibitory receptors including PD-1 and TIM-3 together with reduced production of effector cytokines like IFN-γ (44). In parallel, regulatory T cells exhibit enhanced stability and suppressive function following β-AR activation (47). Collectively, these mechanisms point to the sympathetic–β-AR axis as a critical molecular bridge connecting chronic stress to immune escape by tumors.

By contrast, the role of α2-adrenergic receptors appears more complex and dependent on context. Certain studies suggest that α2-AR agonists may increase macrophage phagocytic activity and trigger apoptosis in MDSCs, thereby alleviating immunosuppression under specific conditions (48). Nevertheless, the precise contribution and cell-type specificity of α2-AR signaling in CRC remain to be fully clarified.

### 3.2 Parasympathetic and cholinergic signaling: context-dependent dual immunoregulation

Parasympathetic regulation of intestinal immunity occurs mainly through vagal and cholinergic pathways. Studies conducted in non-malignant intestinal and inflammatory settings have shown that neural and cholinergic circuits can regulate macrophages and maintain intestinal regulatory T-cell niches (49). These findings provide a physiological basis for cholinergic immune regulation in the gut, but they do not demonstrate an equivalent antitumor or protumor function in CRC. The direction of cholinergic signaling in CRC is therefore likely to depend on disease stage, receptor subtype and cellular context. Although this mechanism may attenuate inflammation-associated tumorigenesis during early stages, it might simultaneously compromise the subsequent activation of effective antitumor immune responses.

Of note, the immune system itself represents a significant source of cholinergic signaling. For instance, T cells expressing choline acetyltransferase (ChAT^+^) can synthesize acetylcholine, which then acts in an autocrine or paracrine fashion to regulate T cell homeostasis and activation thresholds, thereby preventing excessive exhaustion (50). This arrangement constitutes an intrinsic immune regulatory circuit. Furthermore, depending on the cellular context and microenvironmental cues, muscarinic acetylcholine receptors can mediate divergent effects, with certain receptor subtypes being implicated in either pro-inflammatory or immunosuppressive processes—underscoring the complex role of cholinergic signaling in tumor progression (49).

### 3.3 Sensory neural signaling: the convergence of perception and immune regulation

Beyond transmitting nociceptive and environmental stimuli, sensory neurons serve as important regulators of immune responses (51). Sensory neurons can regulate antitumor immunity through the release of neuropeptides, although most causal evidence currently derives from tumor models other than CRC. CGRP can suppress cytotoxic lymphocyte function in selected experimental tumor settings through receptor-dependent signaling (52). These findings identify CGRP as a candidate neuro–immune mechanism relevant to CRC, but direct functional validation in CRC remains limited.

Substance P–NK1R and vasoactive intestinal peptide signaling have been associated with inflammatory and immunoregulatory responses in several experimental systems (53, 54). However, their direct effects on dendritic-cell maturation, PD-L1 expression and regulatory T-cell differentiation in CRC have not been established. These pathways should therefore be considered candidate mechanisms requiring CRC-specific validation.

### 3.4 Neurotrophic factors: linking neural remodeling to immune regulation

Linking structural remodeling of nerves to functional regulation of immunity, neurotrophic factors serve as crucial molecular intermediaries. Secreted by CRC cells along with other components of the tumor microenvironment, neurotrophic signals such as NGF and BDNF activate receptors including TrkA and p75^NTR^. Directed neural growth and infiltration into tumor regions are promoted by these pathways, which also directly impact immune cell behavior.

As a specific example, NGF and BDNF are capable of modulating macrophage polarization while boosting MDSC-mediated suppression, thereby indirectly attenuating effector T cell responses (55). Thus, neurotrophic factors act both as architects of tumor-associated neural networks and as regulators of immune suppression, strengthening positive feedback loops that unify neural remodeling, immune dysregulation, and tumor progression.

## 4 Immune-mediated feedback regulation of the nervous system

Pronounced bidirectionality characterizes neuro–immune interactions within the CRC microenvironment. Immune cells do not merely follow neural instructions; instead, they actively remodel both the structure and function of neurons and glial cells by secreting neurotransmitter-like molecules, bioactive metabolites, and various cytokines. Through such mechanisms, the nervous system becomes deeply embedded in the tumor microenvironment’s dynamic remodeling network, thereby forming a tightly coupled bidirectional regulatory circuit.

### 4.1 Immune cell–derived neuromodulatory mediators

A growing body of evidence suggests that several immune cell types are capable of synthesizing and releasing classical neurotransmitters together with neuromodulatory molecules, thus eroding the conventional boundaries separating the nervous and immune systems. Cholinergic T cells offer a notable example. By expressing choline acetyltransferase, these cells generate acetylcholine, which then acts in an autocrine or paracrine fashion to regulate T cell receptor signaling, metabolic states, and exhaustion trajectories (50). Within CRC, such mechanisms may contribute to local homeostasis; nevertheless, excessive activation of cholinergic pathways might also indirectly dampen robust effector responses, potentially compromising the efficacy of antitumor immunity.

Furthermore, B cell-derived γ-aminobutyric acid (GABA) can engage GABA receptors present on macrophages and other immune cells, driving their differentiation toward anti-inflammatory or suppressive phenotypes (38). This process indirectly impairs CD8^+^ T cell function and aids the establishment of an immunosuppressive microenvironment (39). Upon platelet activation, large quantities of serotonin are released, exerting multifaceted effects that include modulation of neural activity and induction of PD-L1 expression on tumor cells via receptor-mediated signaling, thereby directly driving T cell exhaustion (37). In contrast, dopamine appears to exert more favorable immunological actions. Through engagement of dopamine D5 receptors on CD8^+^ T cells, dopamine signaling promotes the differentiation of tissue-resident memory T cells, consequently enhancing long-term local immune surveillance (40). Taken together, these observations highlight the context-dependent and functionally complex nature of immune-derived neural signals.

### 4.2 Cytokine-mediated regulation of neuronal activity and neural plasticity

Beyond neurotransmitter-like mediators, cytokines produced by immune cells can directly influence neuronal and glial function, modulating excitability, growth, and network remodeling. Such regulation is especially critical within the tightly coupled neuro–immune environment of the gut. For example, IL-17A derived from Th17 cells has been shown to promote neuronal repair and axonal regeneration, potentially through modulation of neuronal survival signaling and local inflammatory cues (56). In the setting of CRC, this neuroregenerative effect might inadvertently support the maintenance and expansion of tumor-associated neural networks.

Concurrently, soluble factors such as leukemia inhibitory factor and granulocyte–macrophage colony-stimulating factor—produced by tumor cells as well as immune cells—can act on peripheral nerves or vagal afferent fibers to transmit local inflammatory signals to the central nervous system, thereby activating regulatory centers including the hypothalamus (25). This “peripheral immune signaling–central neural response” mechanism allows localized tumor inflammation to be amplified into systemic neuroendocrine outputs, establishing feedback loops that continuously shape the tumor microenvironment (57).

### 4.3 Immunoregulatory functions of peripheral glial cells: the case of Schwann cells

Within the peripheral nervous system, glial cells—Schwann cells in particular—serve as crucial regulatory hubs that connect neural with immune compartments. Schwann cells can function as neuro–immune intermediaries in peripheral tissues and in several cancer types. Tumor-associated Schwann cells have been reported to release chemokines such as CCL2 and to participate in neural remodeling and perineural invasion (58). Schwann cells may also express antigen-presenting molecules under selected conditions (59). However, the contribution of these mechanisms to immune regulation in CRC remains insufficiently characterized.

Of note, the capacity of Schwann cells to modulate immune responses displays a dual nature. Schwann cells, guided by signals from the microenvironment, can either drive tumor progression or—given particular circumstances—aid antitumor immunity. Tumor stage, the inflammatory setting, and the fluctuating equilibrium between neural and immune signals inside the tumor microenvironment collectively decide the final result (60).

## 5 Therapeutic strategies targeting neuro–immune interactions

**Figure 3:**
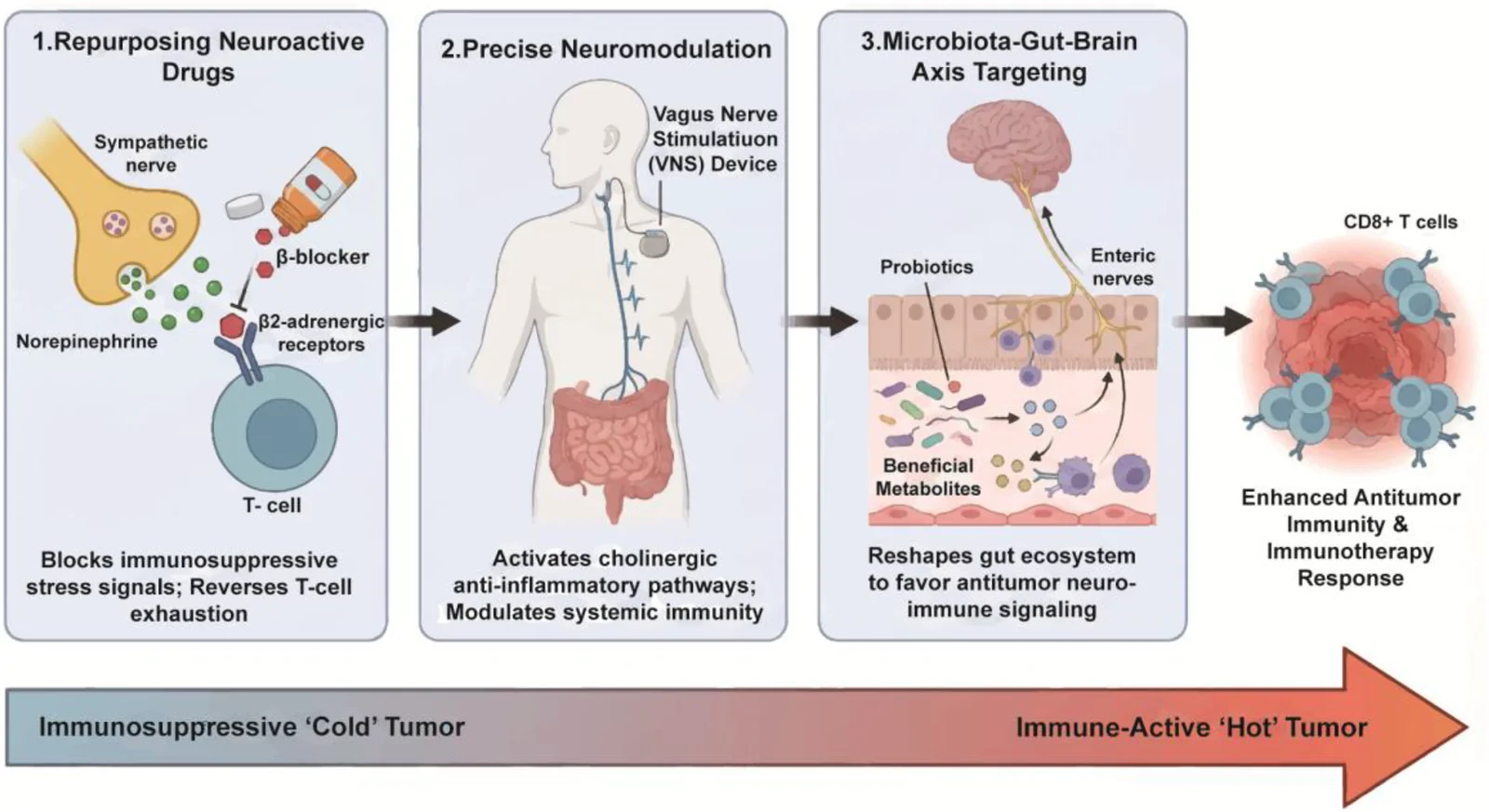
Therapeutic strategies targeting the neuro–immune axis to enhance antitumor immunity in colorectal cancer. Pharmacological blockade of sympathetic stress signaling, precise neuromodulation, and microbiota–gut–brain axis interventions converge to remodel the tumor immune microenvironment. Together, these approaches promote the transition from an immunosuppressive “cold” tumor state to an immune-active “hot” tumor characterized by enhanced CD8^+^ T cell infiltration and improved immunotherapy responsiveness. Created in BioRender. Zhao, M. (2026) https://BioRender.com/3ydmgfq.

Given the growing recognition of neuro–immune crosstalk as a critical driver of colorectal cancer (CRC) initiation and progression (61), approaches directed at neural signaling or its interface with immune pathways are increasingly seen as promising means to reverse immunosuppression and circumvent resistance to immunotherapy. **Table 2** summarizes current therapeutic strategies aimed at neuro-immune interactions, along with their mechanisms and translational status. An integrated depiction of these strategies—targeting the neuro-immune axis and their convergent effects on antitumor immunity—is provided in **Figure 3**. Compared with *de novo* drug development, repurposing existing neuroactive agents for clinical use, directly modulating neural circuits, and combining interventions that involve the gut microbiota all offer clear mechanistic justifications and relatively high translational feasibility.

**Table 2:** Therapeutic strategies targeting the neuro–immune axis in colorectal cancer.

Target axis	Intervention	Immune effect	Evidence level	Therapeutic potential
Sympathetic–β-AR	β-blockers	Restore CD8^+^ T cell function	CRC/preclinical and early clinical (62–67)	Combination with ICIs
Vagal–cholinergic	Vagus nerve stimulation	Modulate inflammation	Non-cancer/preclinical (49, 73, 74)	Neuromodulation strategy
CGRP signaling	CGRP receptor antagonists	Enhance cytotoxic immunity	Non-CRC preclinical (52, 68, 69)	Borrowed from migraine therapy
NGF–Trk	Trk inhibitors	Reduce axonogenesis	Mainly non-CRC preclinical (33, 41, 55)	Prevent neural infiltration
Microbiota–neural	Microbiota modulation	Indirect immune activation	Indirect/emerging (80–85)	Gut-specific advantage
Neural density	Nerve quantification	Risk stratification	Correlative (16, 28, 29)	Prognostic biomarker

### 5.1 Clinical repurposing of neuroactive drugs: from adjunctive therapy to precision immunomodulation

Repurposing approved neuroactive drugs stands out as one of the most translationally attractive strategies (62). Among such agents, β-adrenergic receptor blockers have received the most extensive investigation. In preclinical models, the non-selective β-blocker propranolol has been found to enhance CD8^+^ T cell infiltration, suppress MAPK signaling intrinsic to tumors, and improve the immunological landscape of the tumor microenvironment (63, 64). When administered together with immune checkpoint inhibitors, propranolol further synergizes to boost antitumor immunity, suggesting that targeting the sympathetic–β-adrenergic axis might offer a way to overcome resistance to immunotherapy (65, 66). Nevertheless, clinical outcomes remain variable, highlighting the need for more refined patient stratification (67).

Nevertheless, clinical outcomes remain variable, highlighting the need for more refined patient stratification (68). For example, CGRP receptor antagonists (rimegepant), currently used to treat migraine, have been shown to enhance T cell function in tumor models (52, 69). Similarly, aprepitant—an antagonist of the substance P–NK1R pathway—exhibits inhibitory effects on both tumor growth and metastasis (53, 70). These agents introduce new opportunities for intervening in sensory neuron-mediated immunosuppression.

In addition, selective serotonin reuptake inhibitors (SSRIs), widely prescribed for psychiatric disorders, have been reported to reduce PD-L1 expression on tumor cells by blocking serotonin signaling and to reverse T cell exhaustion. Fluoxetine serves as one such example (37, 71). These observations expand the potential use of neuropsychiatric medications in the modulation of antitumor immunity.

### 5.2 Neuromodulation of neural circuits: from systemic intervention to precise control

In addition to pharmacological strategies, directly modulating neural activity offers another therapeutic avenue (72). Through activation of the cholinergic anti-inflammatory pathway, vagus nerve stimulation—a relatively mature non-pharmacological intervention—regulates systemic immune responses and has been shown in certain tumor models to enhance antitumor immunity, thereby providing insights relevant to potential clinical translation (73, 74).

More advanced methodologies include optogenetic and chemogenetic technologies, which permit high-precision control over specific tumor-associated neural subsets—for instance, sympathetic or sensory neurons—within experimental settings (75, 76). These techniques not only alleviate tumor-related pain but also directly reverse local immunosuppressive states (77). Although their immediate applicability in clinical practice remains limited, they are invaluable for identifying pathogenic neural circuits and for validating novel therapeutic targets (6).

### 5.3 Microbiota–neural–immune triadic interventions: integrating gut ecosystem regulation

Given the pivotal role played by the gut microbiota in regulating both the enteric nervous system and local immune responses (78, 79), modulating the neuro-immune axis through microbiota-based strategies represents an integrative therapeutic approach. Specific probiotic strains—such as Akkermansia muciniphila and Bifidobacterium pseudolongum—together with their metabolites (butyrate and inosine) have been demonstrated to improve ENS function, reshape neurotransmitter profiles, and enhance antitumor immunity (80, 81).

A more disruptive and potentially transformative approach involves combining fecal microbiota transplantation with immune checkpoint blockade (82, 83). Improved responses to immunotherapy have been shown with this strategy in melanoma (84). Given the close relationship between CRC and the intestinal ecosystem, therapies that coordinately remodel microbial communities to regulate both neural and immune components possess unique theoretical as well as translational value (85).

### 5.4 Translational challenges and future directions

Despite encouraging mechanistic findings, the clinical translation of neuro–immune interventions remains uncertain. β-Adrenergic blockade illustrates this challenge. Propranolol has been associated with enhanced CD8^+^ T-cell activity and altered tumor signaling in CRC and other experimental settings (63–66). However, much of the available evidence is derived from preclinical models, short-term pharmacodynamic studies or early-phase investigations in cancer types other than CRC (62, 67). Therefore, changes in immune infiltration or signaling biomarkers should not automatically be interpreted as evidence of durable clinical benefit in patients with CRC.

Several factors may contribute to heterogeneous therapeutic outcomes. First, β-adrenergic receptors are not functionally interchangeable. β1- and β2-adrenergic signaling may differ according to receptor distribution, target-cell type and tissue context, whereas non-selective β-blockers can simultaneously affect cardiovascular physiology, tumor cells, stromal cells and multiple immune populations (45, 46, 63). Second, the biological effect may vary according to tumor stage, treatment timing, exposure duration, local neural density, receptor expression and concomitant anticancer therapy. These variables complicate comparisons among preclinical studies, perioperative interventions and long-term clinical use (67, 86–88).

Similar limitations apply to SSRIs, neuropeptide receptor antagonists and direct neuromodulatory interventions. These approaches may affect platelet activity, vascular function, intestinal motility, neural signaling, immune-cell function and tumor-cell behavior simultaneously, making it difficult to attribute therapeutic effects to a single neuro–immune mechanism (37, 52, 53, 72–74). Furthermore, conventional tumor models do not fully reproduce the enteric nervous system, gut microbiota or spatial organization of tumor-associated nerves in human CRC (86, 89). Future studies should therefore incorporate orthotopic CRC models, spatial profiling, organoid-based neural–immune systems and biomarker-guided patient stratification (90, 91). At present, neuroactive interventions should be regarded as investigational adjuncts rather than established strategies for overcoming immunotherapy resistance in CRC.

## 6 Conclusions and outlook

A fundamental redefinition of the nervous system’s role in colorectal cancer (CRC) has emerged from recent evidence: rather than serving as a passive background regulator, it now stands as an active and integral element of the tumor microenvironment (6). Through an intricate network comprising neurotransmitters, neuropeptides, neurotrophic factors, and immune-derived neuroactive mediators, inputs from sympathetic, parasympathetic, and sensory nerves dynamically engage with immune cells (92). Collectively, these signals orchestrate the recruitment, functional differentiation, metabolic programming, and effector capacity of immune cells, thereby influencing tumor progression, immune evasion, and therapeutic resistance (61).

A critical framework for understanding the immunologically “cold” phenotype seen in the majority of CRCs is supplied by this neuro-immune axis. Limited efficacy of immune checkpoint blockade within this malignancy finds mechanistic explanation through the same axis as well (93). By systematically folding neural regulation into the field of tumor immunology, the present perspective transcends immune-centered models and highlights the nervous system as a determinant of immune competence operating within the tumor microenvironment (94).

Looking ahead, dissecting the causal and context-dependent nature of neuro-immune interactions remains a major challenge (86). To delineate the spatiotemporal architecture of neural–immune crosstalk in cancer, advances in single-cell and spatial multi-omics, *in vivo* neural imaging, and circuit-specific manipulation will be essential (90). Progress in translation will require a shift from generalized neuromodulation toward interventions that are subtype- and circuit-specific, supported by the development of reliable neuro-immune biomarkers for patient stratification and response prediction (87).

Importantly, targeting neuro-immune interactions ought to be viewed as a complementary strategy rather than a standalone approach. Rational integration with existing modalities—including immune checkpoint inhibition, chemotherapy, and radiotherapy—may provide a practical path to overcoming resistance and improving therapeutic efficacy (65). Ultimately, progress in this field will depend on deep interdisciplinary collaboration across neuroscience, immunology, oncology, and bioengineering (95). As these efforts converge, interventions guided by neuro-immune principles are poised to become an essential component of precision therapy in colorectal cancer.
